# Voriconazole-induced lentiginosis in a child: a phototoxicity warning sign

**DOI:** 10.1016/j.abd.2026.501355

**Published:** 2026-05-02

**Authors:** Felipe Henríquez, Vicente Orellana-Westermeyer, Amany Chaaban, Paula Muñoz, Camila Downey, María Teresa Dossi

**Affiliations:** a Department of Dermatology, Faculty of Medicine, Universidad de Chile, Santiago, Chile; b Department of Health Hualañé, Hualañe, Chile; c Faculty of Medicine, Universidad del Desarrollo, Santiago, Chile; d Department of Dermatology, Hospital Luis Calvo Mackenna, Santiago, Chile

Dear Editor,

Voriconazole, a triazole antifungal, is widely used in children with invasive fungal infections, especially in the context of hematologic malignancies and transplantation. Despite its efficacy, long-term therapy has been increasingly associated with phototoxic reactions, lentiginosis, and accelerated Non-melanoma Skin Cancer (NMSC) and melanoma.^1^, ^2^, ^3^ Because the skin is one of the main target organs for the adverse effects of this drug, we present a case that illustrates one of these manifestations and illustrates the importance of early recognition.

A 7-year-old boy with acute lymphoblastic leukemia, atopic dermatitis, and autism spectrum disorder was started on oral voriconazole in August 2023 for an angioinvasive fungal infection. After several months of therapy, he developed multiple, asymptomatic, well-demarcated hyperpigmented macules (2–5 mm) symmetrically distributed on the cheeks, nasal dorsum, and frontal region (Figs. 1A–B). No mucosal, palmar, or plantar involvement was present. There was no family history of lentiginosis or syndromic associations. A diagnosis of chronic voriconazole-induced phototoxicity was made. Because antifungal substitution was not feasible, strict photoprotection (SPF 50+, hat, protective clothing) was recommended. At the 6-month follow-up, the lesions persisted without progression or development of new lesions.

**Figure 1:**
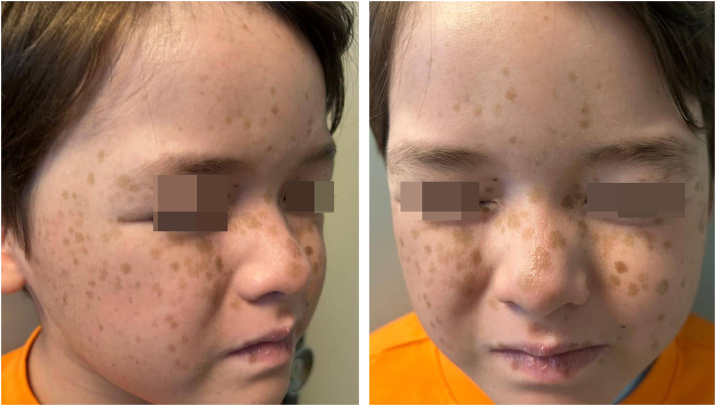
(A and B) 7-year-old boy with multiple well-demarcated hyperpigmented macules symmetrically distributed on the cheeks, nasal dorsum, and frontal region.

Voriconazole-induced phototoxicity occurs in 17%–36% of children, particularly with cumulative exposure or long-term use.^1^, ^2^ Manifestations range from erythema and lentiginosis to premalignant lesions and aggressive Squamous Cell Carcinoma (SCC).^3^ The persistence of lentiginous macules, as in this case, is a marker of chronic UV-induced damage and may represent a potential precursor to malignant transformation. It's also important to make an adequate differential diagnosis of lentigines localized on the head, neck, and acral to exclude conditions such as Peutz-Jeghers syndrome, Laugier-Hunziker syndrome, Cowden syndrome, centrofacial lentigines, and inherited patterned lentigines (Table 1).^4^, ^5^

**Table 1:** Differential diagnosis of localized multiple lentiginosis.

Head and neck involvement	Other localizations
Peutz-Jeghers syndrome	Genital involvement: Bannayan-Riley-Ruvalcaba syndrome
Laugier-Hunziker syndrome	
Cowden disease	Photodistribution: Xeroderma pigmentosum
Centrofacial lentiginosis	Segmental: Partial unilateral lentiginosis
Inherited patterned lentiginosis	

Pathogenesis remains incompletely understood. Proposed mechanisms include the UVA-absorbing properties of voriconazole’s N-oxide metabolite, generating reactive oxygen species that induce oxidative DNA injury.^6^ Additionally, a retinoid-like effect mediated by disrupted retinoic acid metabolism has been hypothesized to contribute to both phototoxicity and keratinocyte changes.^6^

Children represent a particularly vulnerable population due to their prolonged life expectancy, frequent exposure to immunosuppressive regimens, and the need for extended antifungal prophylaxis. Several reports have documented the rapid development of SCC in pediatric transplant recipients under chronic voriconazole therapy.^3^, ^7^ Recent pharmacovigilance analyses by regulatory agencies reaffirmed the importance of phototoxicity surveillance in children treated with voriconazole.^8^

Management includes early dermatologic evaluation, patient and caregiver counseling on rigorous photoprotection, and long-term cutaneous follow-up. Substitution with alternative antifungals such as posaconazole or isavuconazole may be considered in cases of recurrent or severe toxicity, although clinical circumstances may limit this option.

Voriconazole can induce chronic phototoxicity and lentiginosis in children receiving prolonged therapy. It is important for the dermatologist to be familiar with the adverse effect profile of this drug in order to make an adequate differential diagnosis and establish appropriate management, which should include photoprotection and follow-up to identify potential skin neoplasms early.

## ORCID ID

Felipe Henríquez: 0009-0000-4315-6169

Vicente Orellana-Westermeyer: 0009-0007-1507-1863

Amany Chaaban: 0009-0007-8369-910X

Paula Muñoz: 0000-0003-2676-7464

Camila Downey: 0000-0002-1624-1170

María Teresa Dossi: 0009-0008-6838-2881

## Research data availability

Does not apply.

## Financial support

None declared.

## Authors' contributions

Felipe Henríquez: Manuscript writing and editing; data analysis; approved the final version of the manuscript.

Vicente Orellana-Westermeyer: Manuscript writing and editing; data analysis; approved the final version of the manuscript.

Amany Chaaban: Manuscript writing and editing; revision of the manuscript; approved the final version of the manuscript.

Paula Muñoz: Manuscript writing and editing; Supervision of the project; approved the final version of the manuscript.

Camila Downey: Manuscript writing and editing; supervision of the project; approved the final version of the manuscript.

## Conflicts of interest

None declared.
